# Supplementary material for: Downsizing of animal communities triggers stronger functional than structural decay in seed-dispersal networks
Source: Nat Commun. 2020 Mar 27;11:1582. doi: 10.1038/s41467-020-15438-y (PMC7101352; doi:10.1038/s41467-020-15438-y)
Supplement: Supplementary file 1 — Supplementary Information [file 41467_2020_15438_MOESM1_ESM.pdf]

Supplementary Information

Downsizing of animal communities triggers stronger functional than structural decay in seed-dispersal networks

Donoso et al.

Supplementary Table 1 | Geographical description of the eight interaction networks sampled across the Andes.

Network	Code	Latitude	Longitude	Elevational range (m asl)	Sampling years	No. Bird species	No. Plant species	No. of interactions	No. of links	Sampling hours	Source information
Argentina	AR	-26.50	-65.67	1000	2008 - 2010	24	27	1501	105	600	Blendinger et al.[1]
Bolivia	BO	-16.40	-67.50	2500	2010 - 2012	22	19	241	50	768	Saavedra et al.[2]
Colombia 1	CO1	4.71	-75.57	1800	2012	45	22	488	117	600	Muñoz et al.[3]
Colombia 2	CO2	4.70	-75.48	2700	2012	39	26	568	124	600	Muñoz et al.[3]
Ecuador 1	EC1	-3.49	-78.98	1000	2014 - 2015	51	26	1842	204	300	Quitián et al.[4]
Ecuador 2	EC2	-3.97	-79.07	2000	2014 - 2015	36	20	600	79	300	Quitián et al.[4]
Peru 1	PE1	-13.01	-71.54	1500	2009 - 2010	61	52	4988	398	960	Dehling et al.[5]
Peru 2	PE2	-13.17	-71.58	3000	2009 - 2010	26	51	1344	208	720	Dehling et al.[5]

For each of the eight networks, the number of bird species, plant species, total number of observed interaction events and links, and sampling hours are given.

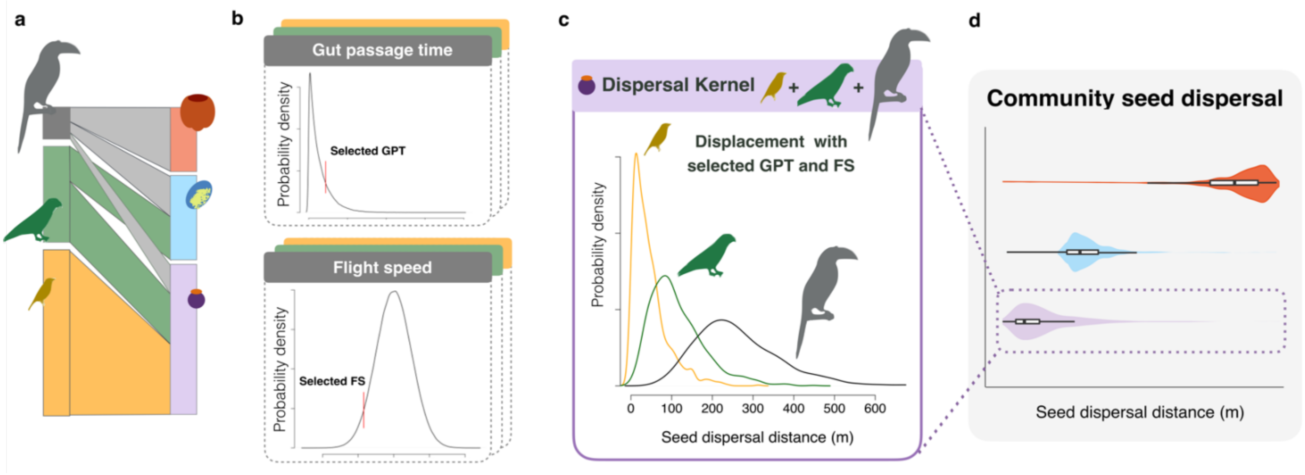

Supplementary Figure 1 | Model framework to link interaction networks and movement ecology to estimate community seed dispersal as a multistage process. (a) Interaction networks provided information regarding the relative contribution of each avian disperser to seed dispersal. **(b)** To estimate the seed dispersal distances provided by each bird species, we applied a trait-based mechanistic seed dispersal model based on allometric scaling relationships between disperser body mass, gut passage time (GPT) and flight speed (FS) (see Methods and Sorensen et al.[6] for details). Values of gut passage time (GPT) and flight speed (FS) were selected from their probability distributions and multiplied to assess the displacement distribution of each bird species. **(c)** For each plant species, dispersal distances from all interaction events across all bird species were combined to define its dispersal kernel (see the example kernel for a single plant species depicted in purple). **(d)** Total community seed-dispersal distance was estimated by combining the dispersal kernels across all plant species in the original network. This figure was modified after Sorensen et al.[6]. Bird and fruit silhouettes by I. Donoso.

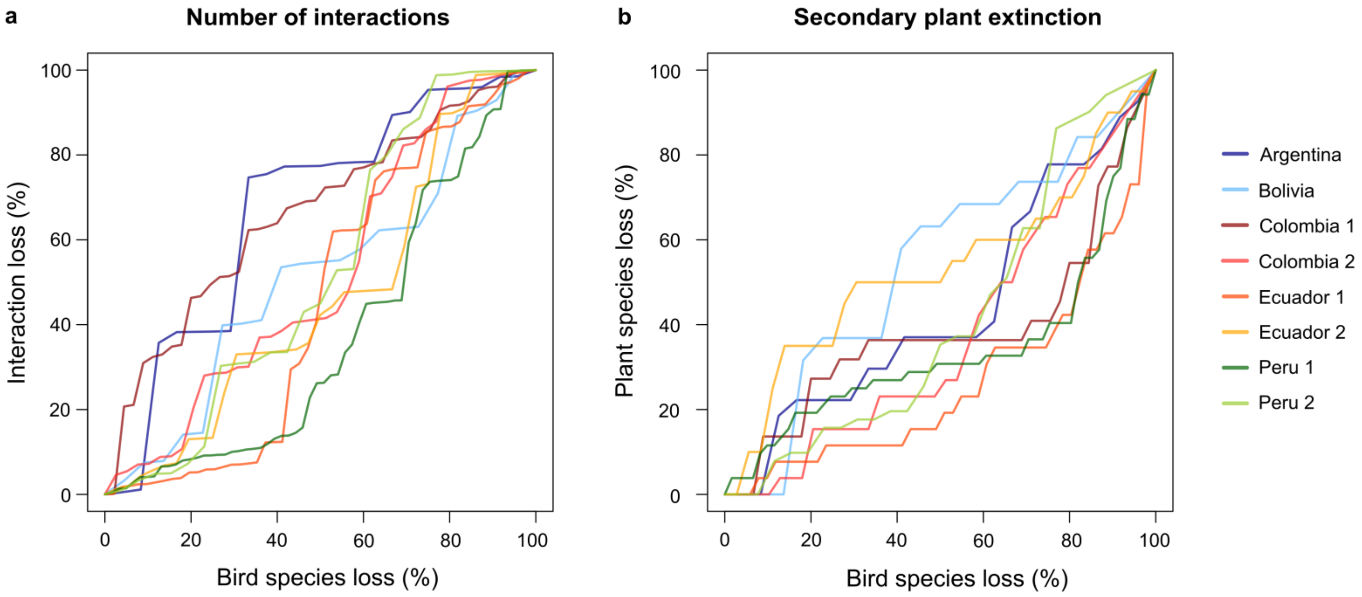

Supplementary Figure 2 | Structural loss in seed-dispersal networks in response to animal extinction. Consequences of size-structured bird extinction on **(a)** the number of interactions and **(b)** secondary plant extinction for each of the eight empirical seed-dispersal networks (depicted in different colours). Values on the y-axis represent the proportional loss relative to the number of **(a)** interactions and **(b)** plant species in each original network.

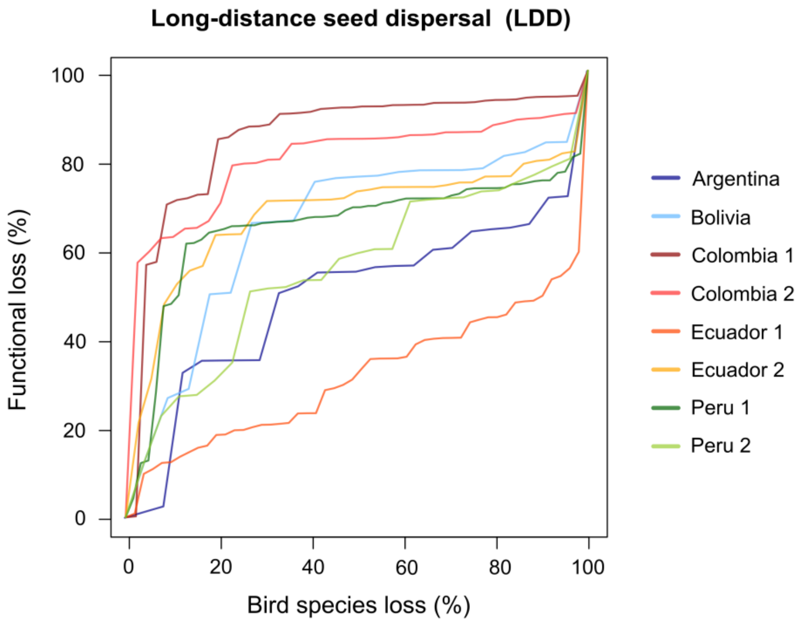

Supplementary Figure 3 | Functional loss in seed-dispersal networks in response to animal extinction. Consequences of size-structured bird extinction on long-distance seed dispersal (LDD) for each of the eight empirical seed-dispersal networks (depicted in different colours). Values on the y-axis show the percentage of functional change defined by the 0.95 quantile of the community-wide seed-dispersal distance relative to the value in each original network.

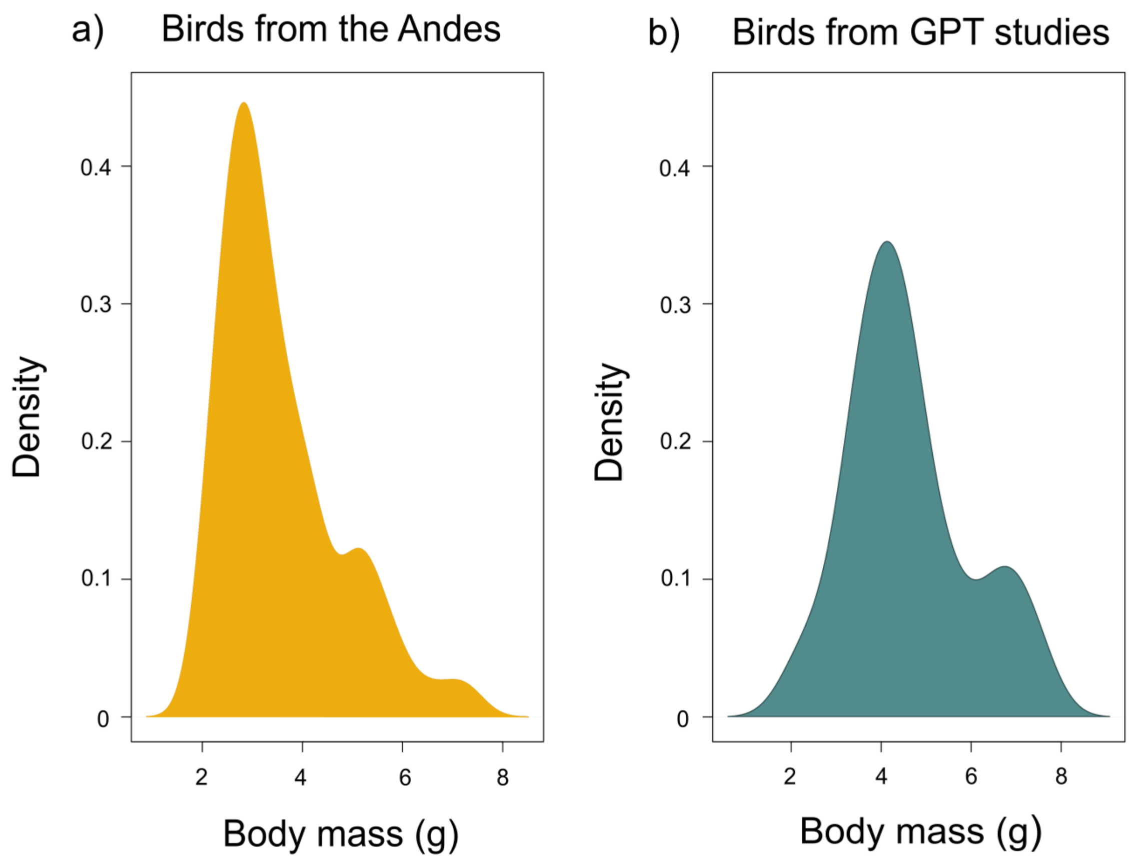

Supplementary Figure 4 | Density plots representing the distribution of bird species' body mass. (a) Body mass distribution of the bird species recorded in the Andean communities and which are provided as Supplementary Data file (n = 179 species). **(b)** Body mass distribution corresponding to the bird species for which the relationship between body mass and gut passage time was available from the literature (n = 34 species, see Table S1 in Sorensen et al.[6] for detailed information about these bird species). Body mass values in **(a)** and **(b)** were transformed to their natural logarithm. We used the *density()* function in R with the default smoothing bandwidth values to create both density plots. Note that the 34 species represent the full range of body mass variation recorded in the Andean communities (body mass ranges: 6.8-1770 g for the Andean species; 9-1770 g for the 34 bird species available in GPT studies, with 97% of the Andean species located in the overlapping zone).

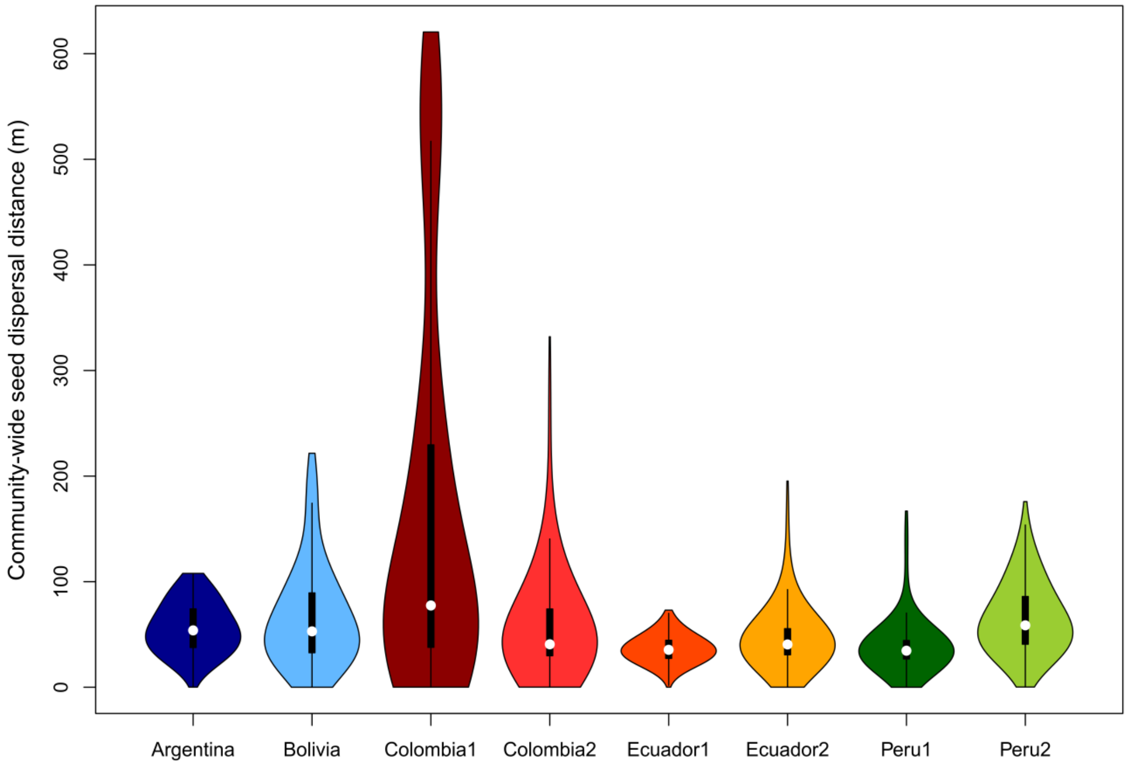

Supplementary Figure 5 | Violin plots representing the seed-dispersal kernel for each of the eight seed-dispersal networks collected across the Andes. Shown are the distributions of the simulated seed dispersal distances (in meters) for the entire plant community of each of the eight empirical seed-dispersal networks (depicted in different colours). The maximum value of each violin plot represents the 0.95 quantile of community-wide seed-dispersal distances (i.e., long-distance seed dispersal).

Supplementary Note 1 | Evaluation of potential regurgitation events on the effect of downsizing on structural and functional losses in seed-dispersal networks.

In this study, we focused on avian seed dispersal events resulting from endozoochory. However, ingested seeds might also be dispersed via regurgitation, which reduces gut passage time (GPT) in comparison to defecation time[7,8]. Regurgitated seeds are thus expected to be dispersed over shorter distances[9]. Previous studies suggested that large seeds, relative to frugivore size, are more likely to be regurgitated[10,11], but that there is no clear allometric relationship between regurgitation time and avian body size[12,8].

We defined defecation vs. regurgitation based on the size of fruits relative to frugivore size[8,13] and specifically used gape width as the most accurate measure of avian size in terms of seed handling[14]. To discard potential regurgitation events from the simulations, we fitted a linear quantile regression (quantile level $\tau = 0.75$) relating fruit diameter and bill width of each interaction event across the full dataset (n = 11,572 events). We assumed that 25% of these events would be due to regurgitation and removed all interaction events located above the fitted regression line (n = 2,889 events, see **Supplementary Figure 6**). Because regurgitation times are known to be shorter than defecation times[7,8], we discarded these events from our simulations because regurgitated seeds would not be relevant for community-wide long-distance seed dispersal. Results of the effect of downsizing on structural and functional losses in seed-dispersal networks were qualitatively identical between simulations including all interaction events and those excluding the potential regurgitation events (**Supplementary Figures 7-9**).

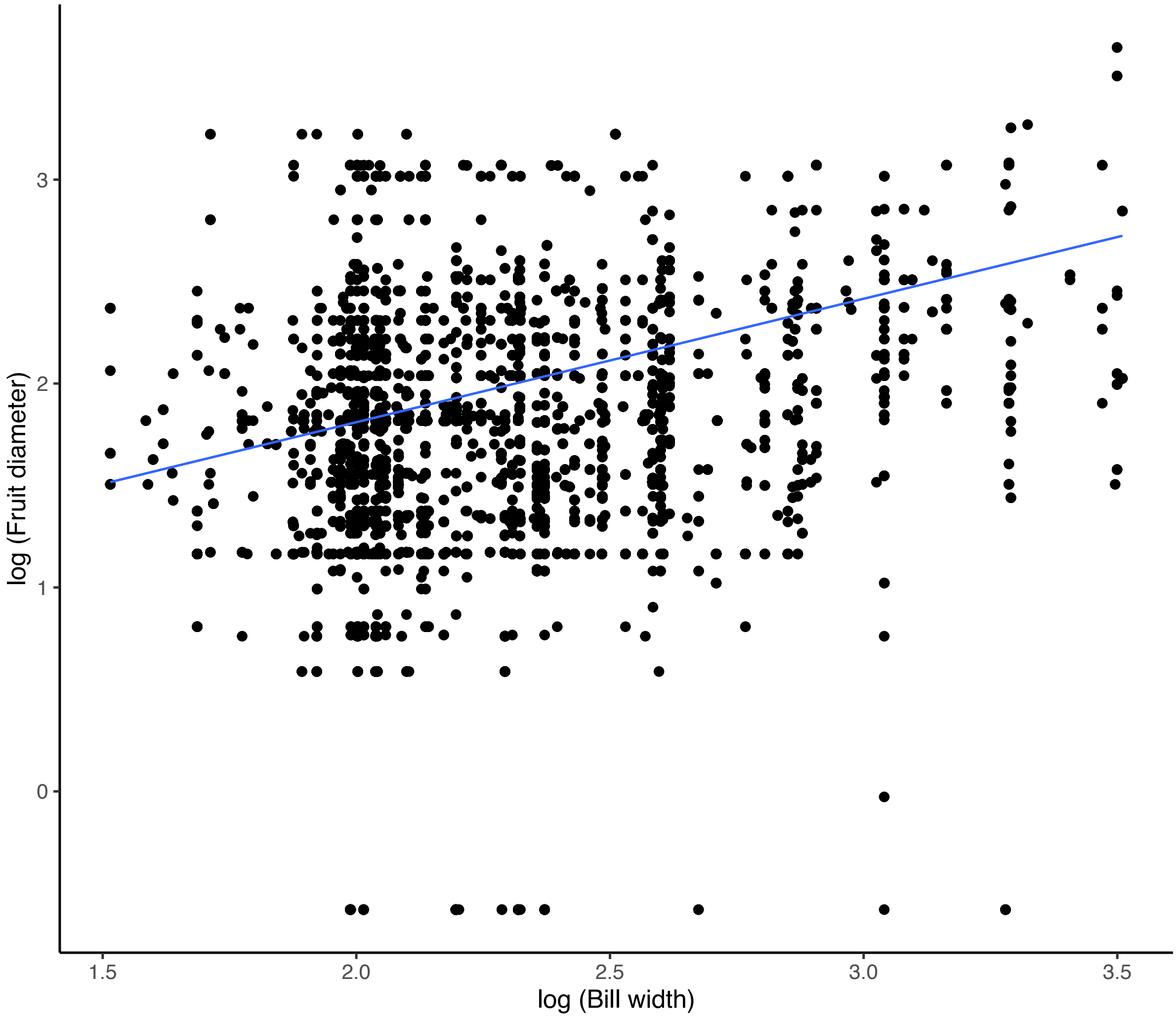

Supplementary Figure 6 | Relationship between avian bill width and fruit diameter. Each black dot represented a plant-frugivore interaction from the eight empirical networks recorded along the Andes. Blue line represented the $\tau = 0.75$ estimate according to the quantile regression model ($\log_{10} D = 0.6 \pm 0.03 + 0.61 \pm 0.014 \log_{10} BW$; parameter estimate ± SE, $p < 0.001$, n = 11,572), which was fitted using the R package *quantreg*[15], version 5.52. We assumed that fruit size in the interaction events above the fitted blue line were large relative to the bill width, so we considered these seed-dispersal events to be likely due to regurgitation and excluded them for the analysis. Results of the effect of downsizing on structural and functional changes after discarding the regurgitated seeds are shown in Supplementary Figs. 7-9.

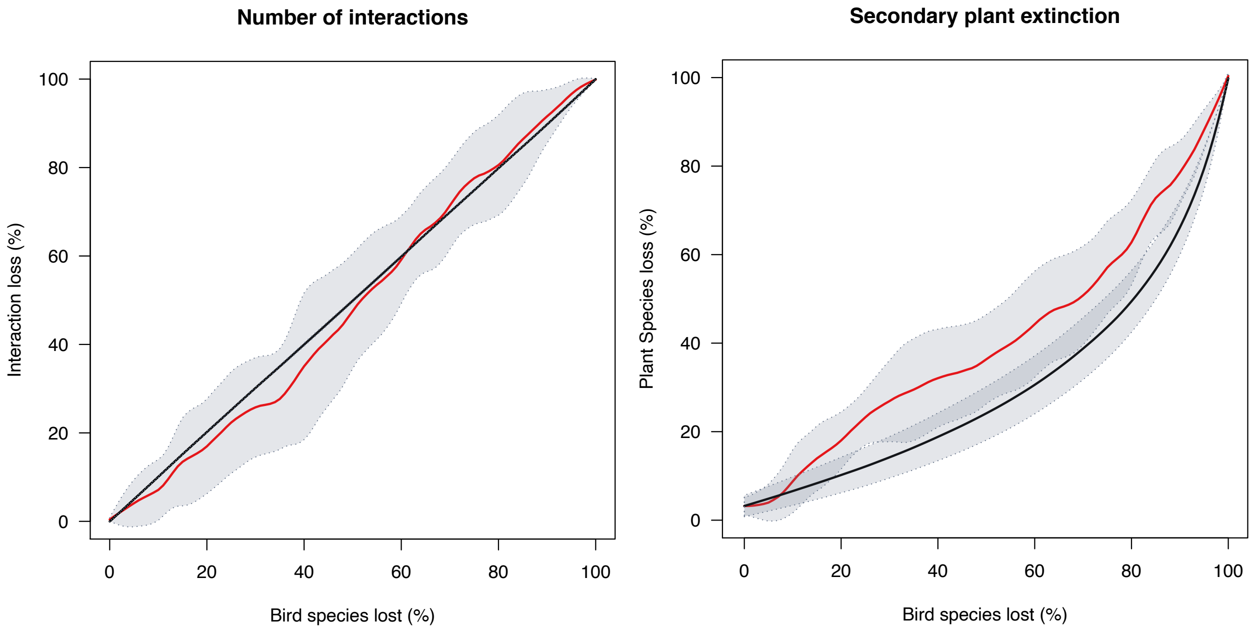

Supplementary Figure 7 | Structural changes in seed-dispersal networks in response to simulated animal extinctions (potentially regurgitated seeds excluded). We compare the consequences of size-structured bird extinctions (downsizing; red line) and random bird extinction (mean across 1000 iterations; black line) on the number of interactions **(left)** and secondary plant species extinctions **(right)**. Values on the y-axis represent the proportional loss relative to the number of interactions and plant species in the original network. Grey areas represent the 95% confidence intervals across the eight Andean seed-dispersal networks. Compare to Fig. 2 showing simulation results including all interaction events.

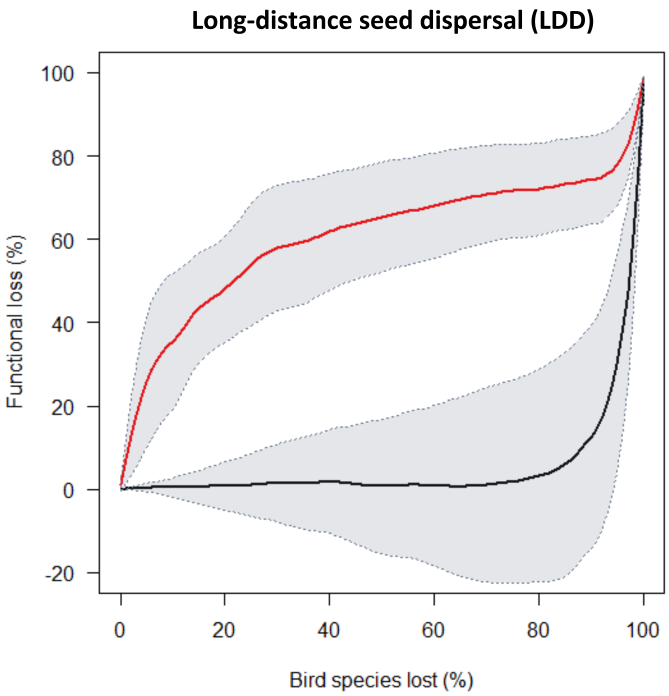

Supplementary Figure 8 | Functional changes in seed-dispersal networks in response to simulated animal extinctions (potentially regurgitated seeds excluded). We compare the consequences of size-structured bird extinctions (downsizing; red line) and random bird extinction (mean across 1000 iterations; black line) on long-distance seed dispersal (LDD). Values on the y-axis show the percentage of functional change defined by the 0.95 quantile of the community seed-dispersal distance relative to the value in the original network. Grey areas represent the 95% confidence intervals across the eight Andean seed-dispersal networks. Compare to Fig. 3 showing simulation results including all interaction events.

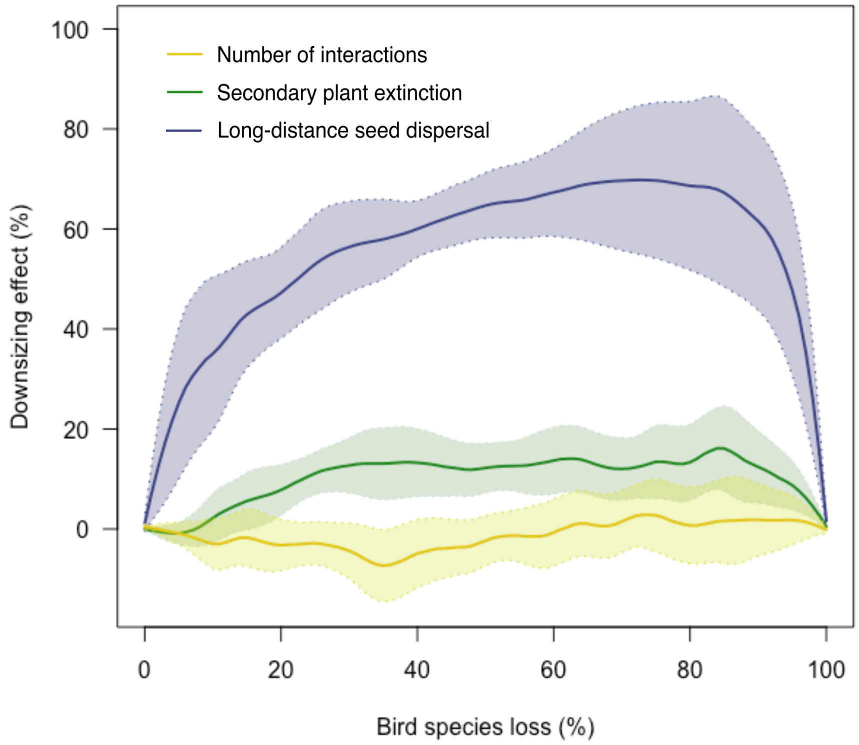

Supplementary Figure 9 | Effect size of downsizing on structural and functional losses in seed-dispersal networks (potentially regurgitated seeds excluded). The effect of downsizing is defined as the difference in the proportional losses between the size-structured and random extinction scenarios. Effect sizes for the number of interactions and secondary plant extinctions (structural loss; yellow and green lines, respectively) are compared to those for long-distance seed dispersal (functional loss; blue line) along the entire extinction sequence. Yellow, green and blue areas represent the 95% confidence intervals across the eight Andean seed-dispersal networks for the effect size of structural and functional losses. Compare to Fig. 4 showing simulation results including all interaction events.

Supplementary References

1. Blendinger, P.G. *et al.* Nutrients in fruits as determinants of resource tracking by birds. *Ibis* **157**, 480-495 (2015).
2. Saavedra, F., *et al.* Functional importance of avian seed dispersers changes in response to human-induced forest edges in tropical seed-dispersal networks. *Oecologia* **176**, 837-848 (2014).
3. Muñoz, C.M., Schaefer H.M., Böhning-Gaese, K. & Schleuning, M. Importance of animal and plant traits for fruit removal and seedling recruitment in a tropical forest. *Oikos* **126**, 823-832 (2017).
4. Quitián, M., *et al.* Elevation-dependent effects of forest fragmentation on plant–bird interaction networks in the tropical Andes. *Ecography* **41**, 1497-1506 (2018).
5. Dehling, D.M. *et al.* Functional relationships beyond species richness patterns: trait matching in plant–bird mutualisms across scales. *Glob. Ecol. Biogeogr.* **23**, 1085-1093 (2014).
6. Sorensen, M. C., Schleuning, M., Donoso, I., Neuschulz, E. L. & Mueller, T. Community-wide seed dispersal distances peak at low levels of specialisation in size-structured networks. Preprint at https://doi.org/10.1101/2020.02.23.958454 (2020).
7. Levey, D. J. Methods of seed processing by birds and seed deposition patterns. In *Frugivores and seed dispersal,* 147-158. (Springer, Dordrecht, 1986).
8. Gasperin, G., & Pizo, M. A. Passage time of seeds through the guts of frugivorous birds, a first assessment in Brazil. *Rev. Bras. Ornitol.*, **20**, 48-51 (2012).
9. Schupp, E. W. Quantity, quality and the effectiveness of seed dispersal by animals. *Vegetatio* **107**, 15-29 (1993).
10. Levey, D. J. Seed size and fruit-handling techniques of avian frugivores. *Am. Nat.*, **129**, 471-485 (1987).
11. Kays, R., Jansen, P. A., Knecht, E. M., Vohwinkel, R., & Wikelski, M. The effect of feeding time on dispersal of *Virola* seeds by toucans determined from GPS tracking and accelerometers. *Acta Oecol.*, **37**, 625-631 (2011).
12. Wheelwright, N. T. How long do fruit-eating birds stay in the plants where they feed? *Biotropica*, **23**, 29-40 (1991).
13. Uriarte, M., Anciães, M., Da Silva, M. T., Rubim, P., Johnson, E., & Bruna, E. M. Disentangling the drivers of reduced long-distance seed dispersal by birds in an experimentally fragmented landscape. *Ecology*, **92**, 924-937 (2011).
14. Dehling, D. M. *et al.* Morphology predicts species' functional roles and their degree of specialization in plant–frugivore interactions. *Proc. R. Soc. B,* **283**: 20152444 (2016).
15. Koenker, R. *quantreg*: Quantile Regression. R package version 5.52. (2019).
