# Supplementary material for: Downsizing of animal communities triggers stronger functional than structural decay in seed-dispersal networks
Source: Nat Commun. 2020 Mar 27;11:1582. doi: 10.1038/s41467-020-15438-y (PMC7101352; doi:10.1038/s41467-020-15438-y)
Supplement: Supplementary file 3 — Reporting Summary [file 41467_2020_15438_MOESM3_ESM.pdf]

nature research

Corresponding author(s): Isabel Donoso

Last updated by author(s): Feb 27, 2020

Reporting Summary

Nature Research wishes to improve the reproducibility of the work that we publish. This form provides structure for consistency and transparency in reporting. For further information on Nature Research policies, see Authors & Referees and the Editorial Policy Checklist .

Statistics

For all statistical analyses, confirm that the following items are present in the figure legend, table legend, main text, or Methods section.

n/a	Confirmed	
☐	☒	The exact sample size (n) for each experimental group/condition, given as a discrete number and unit of measurement
☐	☒	A statement on whether measurements were taken from distinct samples or whether the same sample was measured repeatedly
☒	☐	The statistical test(s) used AND whether they are one- or two-sided<br>*Only common tests should be described solely by name; describe more complex techniques in the Methods section.*
☒	☐	A description of all covariates tested
☐	☒	A description of any assumptions or corrections, such as tests of normality and adjustment for multiple comparisons
☐	☒	A full description of the statistical parameters including central tendency (e.g. means) or other basic estimates (e.g. regression coefficient) AND variation (e.g. standard deviation) or associated estimates of uncertainty (e.g. confidence intervals)
☐	☒	For null hypothesis testing, the test statistic (e.g. F, t, r) with confidence intervals, effect sizes, degrees of freedom and P value noted<br>*Give P values as exact values whenever suitable.*
☒	☐	For Bayesian analysis, information on the choice of priors and Markov chain Monte Carlo settings
☒	☐	For hierarchical and complex designs, identification of the appropriate level for tests and full reporting of outcomes
☐	☒	Estimates of effect sizes (e.g. Cohen's d, Pearson's r), indicating how they were calculated

Our web collection on statistics for biologists contains articles on many of the points above.

Software and code

Policy information about availability of computer code

Data collection	No software was used for data collection.
Data analysis	All analysis were performed in R version 3.4.1.<br>Generalized additive models were performed using the loess() function from the stats package in R (v. 3.4.1).<br>Density plots of Supplementary Figure 4 were plotted using the density() function from the stats package in R (v. 3.4.1).<br>The linear quantile regression in Supplementary Note 1 was fitted using the quantreg R package (v. 5.52).<br>The R code used to estimate community-wide seed-dispersal distance is available as Supplementary Material of the preprint stored at https://doi.org/10.1101/2020.02.23.958454.<br>The Source code of the functions to estimate structural and functional changes under the two extinction scenarios are provided as Supplementary Code 1 (see the executable .txt file "Supplementary_Code_ExtinctionFunctions.txt").

For manuscripts utilizing custom algorithms or software that are central to the research but not yet described in published literature, software must be made available to editors/reviewers. We strongly encourage code deposition in a community repository (e.g. GitHub). See the Nature Research guidelines for submitting code & software for further information.

Data

Policy information about availability of data

All manuscripts must include a data availability statement. This statement should provide the following information, where applicable:

- Accession codes, unique identifiers, or web links for publicly available datasets
- A list of figures that have associated raw data
- A description of any restrictions on data availability

Original data of the eight seed-dispersal networks are stored and metadata are available at the Senckenberg Metacat Data Repository: http://dx.doi.org/10.12761/SGN.2018.10237.
Data on avian body mass for the 179 bird species recorded in the Andean communities are provided as Supplementary Data1. Data on avian body mass was

recorded from the EltonTraits database (Wilman et al. 2014) and are available at http://www.esapubs.org/archive/ecol/E095/178/).

Field-specific reporting

Please select the one below that is the best fit for your research. If you are not sure, read the appropriate sections before making your selection.

☐ Life sciences ☐ Behavioural & social sciences ☒ Ecological, evolutionary & environmental sciences

For a reference copy of the document with all sections, see nature.com/documents/nr-reporting-summary-flat.pdf

Ecological, evolutionary & environmental sciences study design

All studies must disclose on these points even when the disclosure is negative.

Study description	We compiled eight highly resolved, quantitative plant-frugivore networks from the Tropical Andes that have previously sampled by some of the co-authors. All networks are published in previous papers (see details and references in Supplementary Table 1).
Research sample	The data set includes two plant-frugivore networks from Colombia, two networks from Ecuador, two networks from Peru, one network from Bolvia and one network from Argentina. Sampling effort was high in all networks (range of sampling hours: 300-960, mean=606, sd=224), and all networks were collected in near-natural, montane forests. Overall we used data collected during more than 4,800 sampling hours, during which 11,572 events of fruit consumption were recorded. Networks compromised 179 bird species interacting with 227 plant species. These networks only included true seed-dispersal events that correspond to fruit swallowing or carrying-away.<br>Original data of the eight seed-dispersal networks are stored and metadata are available at the Senckenberg Metacat Data Repository: http://dx.doi.org/10.12761/SGN.2018.10237 (2018).<br>Data on avian body mass was recorded from the EltonTraits database, available at http://www.esapubs.org/archive/ecol/E095/178/ (2014)
Sampling strategy	No statistical methods were used to predetermine sample size. The sample size was mainly determined by the availability of high-quality data on plant-frugivore interactions and bird traits. Sampling completeness of each of the eight networks was already proven based on accumulation curves for bird richness and the number of interaction, which showed saturating trends based on the Chaos richness estimator.
Data collection	Interaction network data were compiled by the authors from previous studies (see Data Repository: http://dx.doi.org/10.12761/SGN.2018.10237 (2018) and Supplementary Table 1). We also recorded data on avian body mass from the EltonianTraits database published by Wilman et al. 2014 (Ecology) and available at http://www.esapubs.org/archive/ecol/E095/178/
Timing and spatial scale	The eight networks covered an elevation range from 1000 to 3000 m a.s.l. and extend from 4.7°N to 26.5°S, covering a large portion of the tropical and subtropical Andean range. All networks were sampled to cover both rainy and dry seasons (see sampling years and more details in Supplementary Table 1).
Data exclusions	No data were excluded from the analyses.
Reproducibility	Original data of the eight seed-dispersal networks are stored and metadata are available at the Senckenberg Metacat Data Repository: http://dx.doi.org/10.12761/SGN.2018.10237 .<br>Data on avian body mass was recorded from the EltonTraits database (available at http://www.esapubs.org/archive/ecol/E095/178/).<br>The R code used to estimate community-wide seed-dispersal distance is available as Supplementary Material of the preprint stored at https://doi.org/10.1101/2020.02.23.958454.<br>The Source code of the functions to estimate structural and functional changes under the two extinction scenarios are provided as Supplementary Code 1.
Randomization	There was no randomization procedure related to the dataset. However, we did compare the results derived from a deterministic extinction scenario to those derived from a random scenario (defined by the mean of 1,000 random sequences of bird extinction from the seed-dispersal networks).
Blinding	Not Applicable to our study.

Did the study involve field work? ☐ Yes ☒ No

Reporting for specific materials, systems and methods

We require information from authors about some types of materials, experimental systems and methods used in many studies. Here, indicate whether each material, system or method listed is relevant to your study. If you are not sure if a list item applies to your research, read the appropriate section before selecting a response.

Materials & experimental systems

n/a	Involved in the study
☒	☐ Antibodies
☒	☐ Eukaryotic cell lines
☒	☐ Palaeontology
☒	☐ Animals and other organisms
☒	☐ Human research participants
☒	☐ Clinical data

Methods

n/a	Involved in the study
☒	☐ ChIP-seq
☒	☐ Flow cytometry
☒	☐ MRI-based neuroimaging
