# Supplementary material for: Downsizing of animal communities triggers stronger functional than structural decay in seed-dispersal networks
Source: Nat Commun. 2020 Mar 27;11:1582. doi: 10.1038/s41467-020-15438-y (PMC7101352; doi:10.1038/s41467-020-15438-y)
Supplement: Supplementary file 4 — Description of Additional Supplementary Files [file 41467_2020_15438_MOESM4_ESM.pdf]

Description of Additional Supplementary Files

File Name: Supplementary Data 1
Description: List of the 179 bird species recorded in the Andean communities. The following information are listed: scientific name and family (as for March 2019; based on the IOC World Bird List – version 9.1 – gathered from Avibase - https://avibase.bsc-eoc.org), body mass (from Wilman et al. 2014), and the interaction network(s) in which the species were recorded (see Supplementary Table 1 for the codes of the interaction networks).

File Name: Supplementary Code 1
Description: Source code for the functions to simulate species extinctions in the R language for statistical computing.
